# The complete chloroplast genome sequence and phylogenetic analysis of *Ilex* ‘Beryl’, a hybrid of *Ilex cornuta* × *Ilex latifolia* (Aquifoliaceae)

**DOI:** 10.1080/23802359.2020.1861569

**Published:** 2021-01-20

**Authors:** Fan Zhang, Hong Chen, Yanwei Zhou, Naiwei Li, Xinran Chong, Yunlong Li, Xiaoqing Lu, Chuanyong Wang

**Affiliations:** Jiangsu Key Laboratory for the Research and Utilization of Plant Resources, Institute of Botany, Jiangsu Province and Chinese Academy of Sciences, Jiangsu, China

**Keywords:** *Ilex* ‘Beryl’, complete chloroplast genome, phylogenetic analysis

## Abstract

*Ilex* ‘Beryl’ is an ornamental and ecological tree widespread in southeastern China. In this study, the complete chloroplast (cp) genome of *Ilex* ‘Beryl’ was assembled and characterized to investigate its phylogenetic relationship. The entire cp genome of ‘Beryl’ was a typical quadripartite structure with 157,575 bp in length, including a large single-copy (LSC) region of 87,080 bp and a small single-copy (SSC) region of 18,427 bp, which were separated by a pair of inverted repeats (IRs) of 52,068 bp. There are 135 genes annotated, including 90 protein-coding genes, eight rRNA genes and 37 tRNA genes. Phylogenetic analysis based on whole cp genome sequences showed that ‘Beryl’ is closest to *I.* ‘Emily Bruner’ and *I.* ‘tall boy’.

The *Ilex* L. (holly) is the largest woody dioecious angiosperm genus, containing approximately 700 species within the monogeneric family of Aquifoliaceae (Su et al. 2020). The holly family plants have been wildly cultivated for ornamentals, ecological, and economical purposes. Among them, *Ilex* ‘Beryl’, an artificial hybrid between *I. cornuta* Lindl. and *I. latifolia* Thunb., which has been widely spread in southeastern China for its ornamental, ecological and economical values. However, due to the similar leave with other species and cultivars, it is difficult to be distinguished and identified by morphology (Yao et al. 2016). As an effective DNA molecular marker, the chloroplast genome has been widely used in genetic and evolutionary relationships studies in plants (Freitas et al. 2018). In this work, we obtained the complete sequence of the chloroplast genome of *I.* ‘Beryl’ by using next-generation sequencing technology and analyzed its phylogenetic evolution, which would be helpful for further study on the identification and classification of genus *Ilex*.

DNA samples were collected from the fresh leaves of *I*. ‘Beryl’ in Nanjing Botanical Garden, Mem. Sun Yat-sen (118°49′55″E, 32°3′32″N), Nanjing, China. The voucher specimen (NO. NBGJIB-Ilex-0008) was deposited in the Institute of Botany, Jiangsu Province and Chinese Academy of Science. Total DNA was extracted using the GMS16011.2.1 Kit (Genmed Scientifics Inc., U.S.A). A paired-end library with an insert-size of 350-bp was constructed and sequenced on the Illumina NovaSeq system (Illumina, San Diego, CA). In total, 6039.9 Mb of raw data (5715.8 Mb clean data) were obtained. De novo genome assembly and annotation were conducted by NOVOPlasty (Dierckxsens et al. 2017) and GeSeq (Tillich et al. 2017), respectively. The annotated cp genome was deposited in Genome Warehouse database (accession number: GWHAOTL01000000).

The whole cp genome sequence of *I.* ‘Beryl’ was 157,575 bp (GC content is 37.65%) and has four typical subregions, including a large single-copy (LSC) region of 87,080 bp and a small single-copy (SSC) region of 18,427 bp, which was separated by two inverted repeats (IRs, including IRa and IRb) of 52,068 bp. The ‘Beryl’ cp genome contained 135 annotated genes, including 37 tRNA genes, eight rRNA genes and 90 protein-coding genes. Eight protein-coding genes, four rRNA genes and seven tRNA genes are duplicated in the IR regions. A total of 15 genes contained one (13 genes) or two (*ycf3*, *clpP*) introns.

To determine the phylogenetic status of *I.* ‘Beryl’, 15 other *Ilex* and one Helwingia chloroplast genomes from Aquifoliaceae (Yao et al. 2016; Cascales et al. 2017; Park et al. 2019; Su et al. 2020) were used for constructing maximum likelihood (bootstrap repeat is 1,000) phylogenetic trees using PhyML v3.0 (http://www.atgc-montpellier.fr/phyml/) (Liu et al. 2019). The phylogenetic tree show that ‘Beryl’ is clustered into the *Ilex* section, and has more closely related to hybrid lines of *I.* ‘Emily Bruner’ and *I.* ‘tall boy’ (Figure 1). The cp genome sequence of *I.* ‘Beryl’ in this study will provide an indispensable resource for the conservation genetics of Aquifoliales, and will be useful for further analysis on molecular markers and molecular breeding.

**Figure 1.**
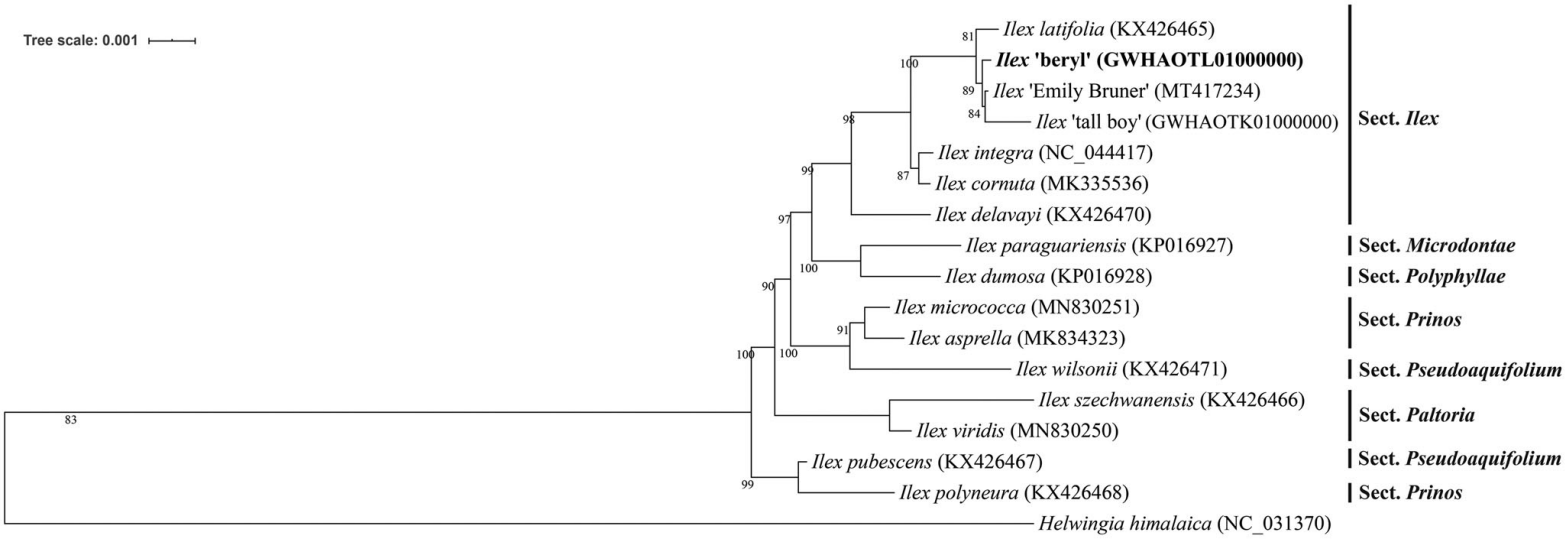
Maximum-likelihood phylogenetic tree based on the sequences of *Ilex* ‘Beryl’ and other 16 complete chloroplast genomes. Section names were displayed in the right side of phylogenetic tree (Su et al. 2020). Numbers on the nodes indicate bootstrap values.

## Data Availability

The complete chloroplast genome sequence of *Ilex* ‘Beryl’ is deposited in the Genome Warehouse (https://bigd.big.ac.cn/gwh) database under the accession number GWHAOTL01000000. The raw sequencing data are deposited in the Genome Sequence Archive database (https://bigd.big.ac.cn/gsa) under the accession number CRA003330.
